# Application of Plackett–Burman Design for Spectrochemical Determination of the Last-Resort Antibiotic, Tigecycline, in Pure Form and in Pharmaceuticals: Investigation of Thermodynamics and Kinetics

**DOI:** 10.3390/ph15070888

**Published:** 2022-07-19

**Authors:** Ahmed S. El-Shafie, Aseel Yousef, Marwa El-Azazy

**Affiliations:** Department of Chemistry and Earth Sciences, College of Arts and Sciences, Qatar University, Doha P.O. Box 2713, Qatar; aelshafie@qu.edu.qa (A.S.E.-S.); ay1604075@student.qu.edu.qa (A.Y.)

**Keywords:** tigecycline, TCNQ, charge transfer reaction, design of experiments (DoE), Plackett–Burman design, pharmaceutical formulation, method validation, thermodynamics, kinetics

## Abstract

Tigecycline (TIGC) reacts with 7,7,8,8-tetracyanoquinodimethane (TCNQ) to form a bright green charge transfer complex (CTC). The spectrum of the CTC showed multiple charge transfer bands with a major peak at 843 nm. The Plackett–Burman design (PBD) was used to investigate the process variables with the objective being set to obtaining the maximum absorbance and thus sensitivity. Four variables, three of which were numerical (temperature—Temp; reagent volume—RV; reaction time—RT) and one non-numerical (diluting solvent—DS), were studied. The maximum absorbance was achieved using a factorial blend of Temp: 25 °C, RV: 0.50 mL, RT: 60 min, and acetonitrile (ACN) as a DS. The molecular composition that was investigated using Job’s method showed a 1:1 CTC. The method’s validation was performed following the International Conference of Harmonization (ICH) guidelines. The linearity was achieved over a range of 0.5–10 µg mL^−1^ with the limits of detection (LOD) and quantification (LOQ) of 166 and 504 ng mL^−1^, respectively. The method was applicable to TIGC per se and in formulations without interferences from common additives. The application of the Benesi–Hildebrand equation revealed the formation of a stable complex with a standard Gibbs free energy change (∆*G°*) value of −26.42 to −27.95 kJ/mol. A study of the reaction kinetics revealed that the CTC formation could be best described using a pseudo-first-order reaction.

## 1. Introduction

**Scheme 1:**
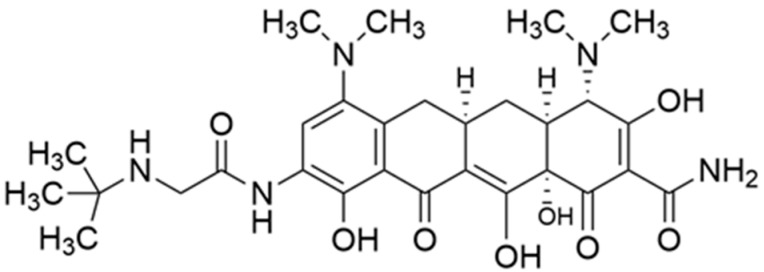
Chemical structure of TIGC.

Tetracyclines (TCs) are a large family of antibiotics that are used both within veterinary and therapeutic rehearsals [1]. Tigecycline (TIGC), as shown in Scheme 1, is the newest member of the tetracycline family that belongs to its third generation [2,3]. TIGC is the first antibacterial drug to be classified under glycylcyclines [4]. Until 2019, TIGC was listed as an ‘essential medicine’ by the World Health Organization (WHO) [5,6]. As a (9-*t*-butylglycylamido) derivative of the parent minocycline, TIGC possesses an enhanced ability to overcome the two resistance mechanisms that the TCs could encounter: ribosomal protection and the TC-specific efflux pump acquisition [7].

In this itinerary, TIGC is a broad-spectrum antibiotic that kills bacterial cells by inhibiting protein synthesis. TIGC is commonly utilized to cure several diseases such as those caused by skin and intra-abdominal pathogens [7]. Yet, first and foremost, TIGC is used to cure infections instigated by multiple-drug resilient pathogens [8]. Recently, TIGC has been considered as a promising candidate for treating acute myeloid leukemia [9]. With an unknown influence on the emergence of resistance in hospitalized patients and low possibilities for renal and other organ toxicity, the use of TIGC could help reduce the hospital burden on the other broad-spectrum antimicrobials [7]. Nevertheless, the administration of TIGC was related to some incidents of unknown deaths. As of 2010, a black box warning for TIGC was consequently released [10]. As a result, the use of TIGC as a last-resort drug was reserved for conditions in which an alternate treatment is not suitable. Moreover, with the release of TIGC into aquatic bodies that likely contain the *Tet(X)* gene, the degradation of this antibiotic possibly causes the further emergence of microbial resistance [2,3,11].

Therefore, finding a simple and sensitive approach for sensing even ultra-low concentrations of TIGC is crucial. A survey of the literature shows that the majority of the efforts in this regard were dedicated to studying TIGC in terms of its mechanism of action, pharmacokinetics, and pharmacodynamics, with fewer efforts being dedicated to finding a simple analytical technique for the determination of TIGC. The reported techniques mainly included chromatographic-based approaches [12,13,14,15,16] and to a lesser extent spectrophotometric [17] and spectrofluorimetric [18,19] approaches. By and large, the chromatographic approaches are sophisticated and require well-trained staff. Moreover, and to the best of our information, all the reported techniques for the analysis of TIGC were univariate-based, where a single variable is investigated at a time while the rest are kept constant. Employing this practice for optimizing process variables implies the absence of the overall vision of the process with a greater consumption of chemicals and resources.

**Scheme 2:**
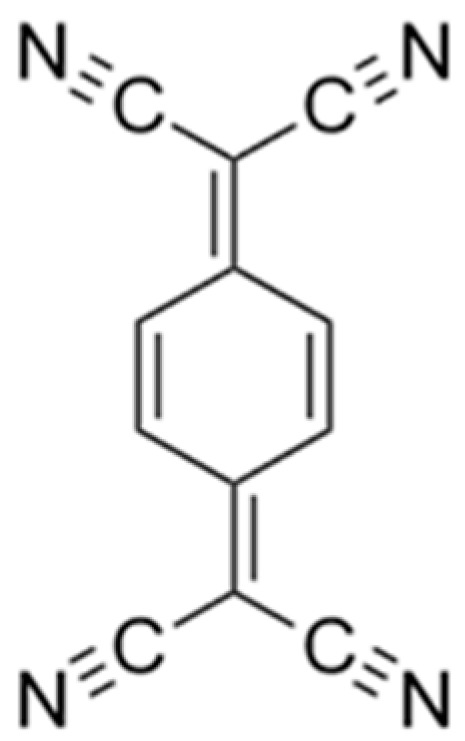
Chemical structure of TCNQ.

Charge transfer complexes (CTC) are usually formed via molecular interactions of electron donors and electron acceptors with the subsequent development of a colored complex that exhibits different behaviors and properties [20,21,22]. 7,7,8,8-tetracyanoquinodimethane (TCNQ) is a strong electron acceptor with four cyano-groups and a π-conjugation system, as shown in Scheme 2 [21].

In the current study, TCNQ will be used as the π-acceptor to react with TIGC as an electron donor to produce an intensely colored CTC. The approach we are offering herein is unique in terms of utilizing CTC for the sensitive and selective determination of TIGC, as well as being the first multivariate-based approach for the determination of TIGC with all the benefits this platform offers. In addition to saving time, efforts, and lowering the possible number of experimentations, a huge amount of data will be generated, an issue that helps to illustrate the reaction under investigation. Therefore, the conclusions can be considered highly irrevocable. It is noteworthy to mention that the Plackett–Burman design (PBD) is a two-level fraction factorial screening design that is used when only the main variables are concerned [23,24,25,26].

The aim of this work is to determine TIGC in its pure form and in the pharmaceutical dosage forms via a sensitive and selective spectrochemical approach. Moreover, the optimum conditions that maximize the reaction response, absorbance in this case, can be realized using PBD as a strategy. In this itinerary, four variables (temperature, reagent volume ‘RV’, reaction time ‘RT’, and the diluting solvent ‘DS’) will be assessed. One response variable will be measured: absorbance at 843 nm (Y_ctc_). Therefore, the optimized approach can be applied in quality control and quality assurance laboratories especially in developing countries where the chance of using sophisticated approaches such as chromatography is not always plausible. Job’s method of continuous variation will be applied to determine the best stoichiometry for the TIGC-TCNQ interaction [27]. The limits of detection (LOD) and quantification (LOQ) will be determined and the whole process will be assessed in terms of accuracy, precision, repeatability, ruggedness, and will follow the International Conference of Harmonization (ICH) recommendations [28].

## 2. Results and Discussion

### 2.1. Absorption Spectra

**Figure 1:**
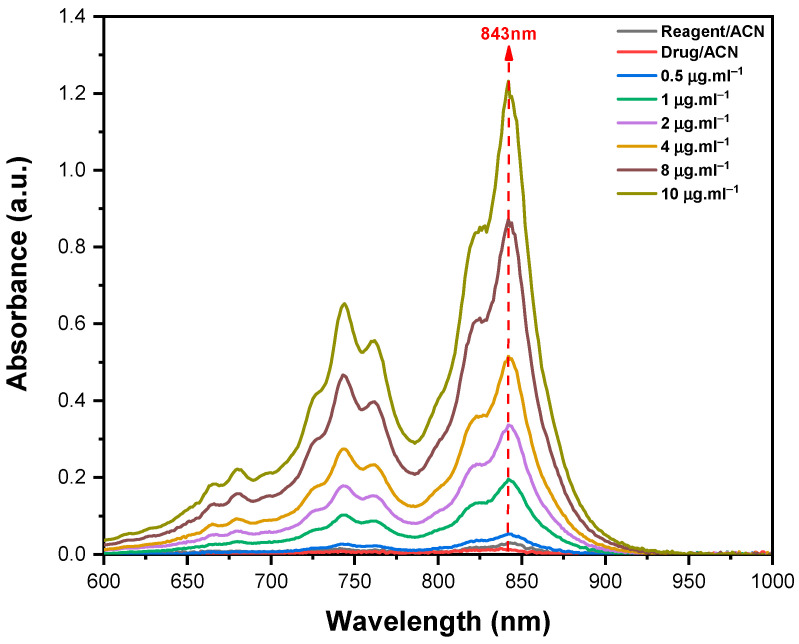
Absorption spectrum for the TIGC-TCNQ CTC obtained under conditions of Temp = 50 °C, RV = 1.5 mL, RT = 60 min, and DS: ACN. Spectra were drawn against a reagent blank (TCNQ prepared in ACN) and a blank of the drug (also prepared in ACN).

The interaction of TCNQ as a π-acceptor with TIGC as an n-electron donor has produced an intensely colored green complex that shows three major peaks at 843, 744, and 680 nm, as shown in Figure 1. These peaks can be ascribed to the development of TCNQ radical anions as a result of the transfer of n-electrons from TIGC to TCNQ in the organic solvent that was used (acetonitrile, ACN). The absorption band at 843 nm was chosen as the analytical wavelength considering the sensitivity and blank absorbance.

### 2.2. Charge Transfer Reaction: Proposed Pathway

**Scheme 3:**
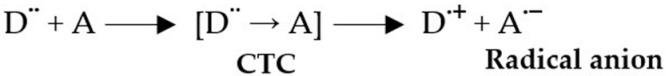
General interpretation of the formation of the CTC.

The interaction of TIGC (D) and TCNQ (A) is probably based on the reaction of the nitrogen atom of TIGC as a donor with the π-acceptor system of TCNQ to produce a CTC. The resulting product will consequently dissociate into radical anions as determined by the solvent polarity. In the current investigation, can the polar solvent, will assist in a complete electron transfer from a TIGC to TCNQ moiety with the formation of intensely colored radical anions, as shown in Scheme 3 [29,30,31,32].

The structure of TIGC shows five ionizable groups: three basic nitrogens and two acidic hydroxyl groups [2,33,34]. In TIGC, the n-electron donation could have occurred via the aliphatic amine group (carboxamide side chain of Ring A), or the secondary amine group in the side chain (pK_a_ = 8.9), or the tertiary amino group at ring A (pK_a_ = 9.5). However, steric hinderance and the unavailability or delocalization of electrons to the benzene ring should be taken into consideration.

### 2.3. Assessment of Reaction Conditions

#### 2.3.1. Plackett–Burman Design (PBD)

As previously revealed, the PBD was the design used to optimize the reaction conditions. Four variables (three numerical and one non-numerical) and a single response variable were considered. The target was set to maximize the response. The PBD generates a number of runs (N) that is a multiple of four.

The PBD is the design of choice when merely the key factors are concerned. The PBD is one of the ordinarily used designs in assessing the robustness while validating an analytical method. The key reason for its use is that the PBD only focuses on the main variables [24,35,36]. The screened factors are shown in Table 1, while the design matrix is shown in Table 2 along with the measured and the predicted responses.

#### 2.3.2. Response Transformation and Modelling

**Figure 2:**
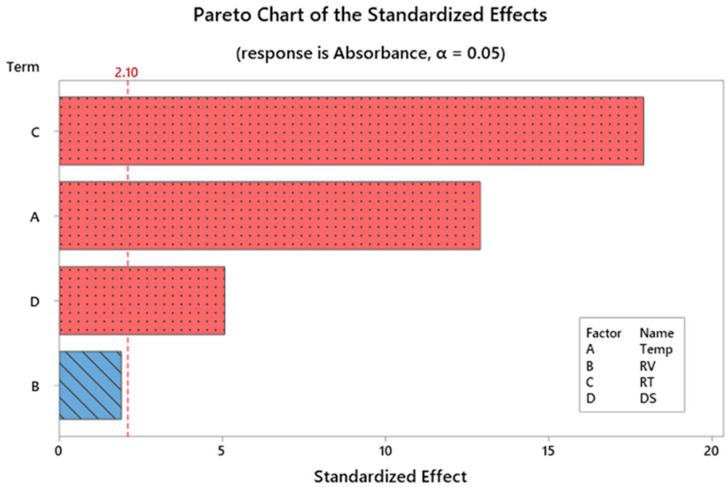
Pareto chart of the studied variables showing the statistical significance of each variable on the absorbance of the colored CTC following response transformation.

The assessment of the substantial variables was performed using different approaches. The Pareto chart of standardized effects was one of the approaches that were used accompanied by an analysis of variance (ANOVA) testing at a 95.0 confidence interval (CI). As shown in Figure 2, variables that exceed the reference line are seen as statistically significant. The reaction time (RT, C) was the most influencing variable while the reagent volume (RV, B) was statistically insignificant. Similar conclusions can be derived from the ANOVA testing shown in Table 3 where only the RV has a probability value (*p*-value) that exceeds the significance level (α = 0.05), implying that the variable is statistically insignificant. Table 3 also shows that the lack-of-fit values were close to 1.000, thus implying the goodness-of-fit of the obtained data.

It is important to point out that these data were collected after response transformation, applying the Box–Cox response transformation where the value of λ (transformation factor) was 0.50 as per the following equation, Equation (1) [37,38]:

(Transformed response) Y′ = (Y_λ_ − 1)/λ (transformation factor) (1)

**Figure 3:**
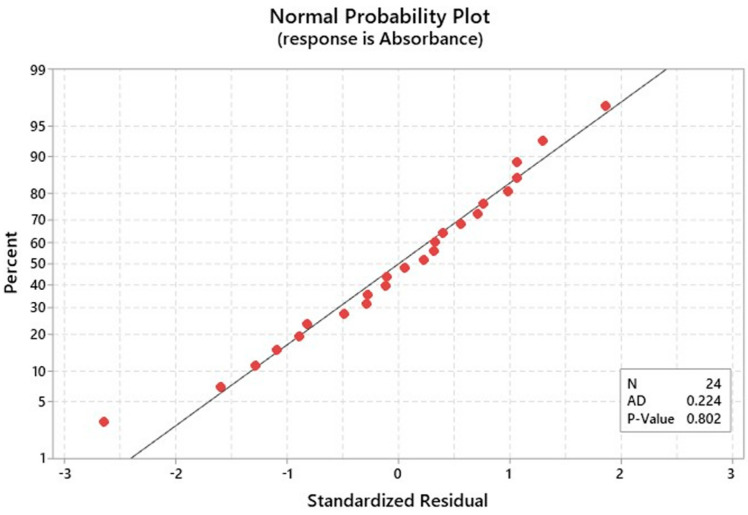
Normal probability plot of residuals.

The output of this processing is the mathematical model shown in Equation (2):

√Y_CTC_ = 0.2463 + 0.007897 Temp + 0.0467 RV + 0.007969 RT + 0.0566 DS − 0.0448 Ct. Pt. (2)

where Ct. Pt. is the central point. The model summary shows the coefficient of determination, R^2^ = 0.9665, R^2^ (adjusted) = 0.9572, and R^2^ (predicted) = 0.9378. The high value of R^2^ reflects the fact that the proposed model well-fit the obtained data. The high value of R^2^ (predicted) infers that the model has a good prediction capability for new observations. This is well-reflected and can be concluded from Table 2, where the values of the predicted absorbance were very close to the observed ones as inferred from the small values of RE. The difference between R^2^ (adjusted) and R^2^ (predicted) was less than 10%, implying the absence of model over-fitting. The normal probability plot of residuals was used to verify the postulation that the residuals are normally distributed. Figure 3 shows that Anderson–Darling (AD) indicator value, which is used to determine how favorably data obey a certain distribution, is less than the *p*-value, signifying the good distribution of the data [39].

#### 2.3.3. Impact of the Diluting Solvent

**Figure 4:**
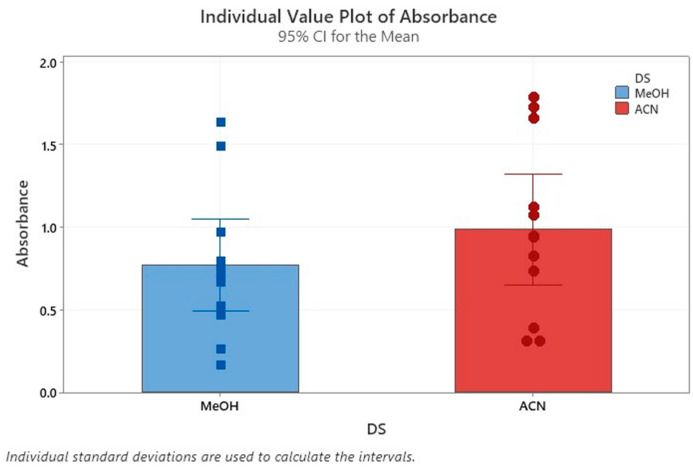
Individual value plots for the measured absorbance of the colored CTC with added interval bars at 95.0% CI.

As a part of the investigation, the impact of the polarity of the diluting solvent on the measured response was investigated. The individual value plot was used to check the presence of outliers and the distribution coverage, as shown in Figure 4. A visual inspection of the represented data shows that the use of ACN has resulted in a slightly higher absorbance compared to methanol (MeOH).

A further investigation was conducted using a 2-sample t-test. The purpose of conducting this test was to measure the effect of the used solvent on the absorbance of the CTC. The obtained results implied that the difference between MeOH and ACN on the CTC that was formed was statistically insignificant, where the *p*-value was greater than the significance level (α = 0.05). In addition, the mean of the measured response in the case of ACN was not markedly higher in comparison to that obtained using MeOH, as shown in Table 4.

#### 2.3.4. Optimization Phase: Contour Plots

**Figure 5:**
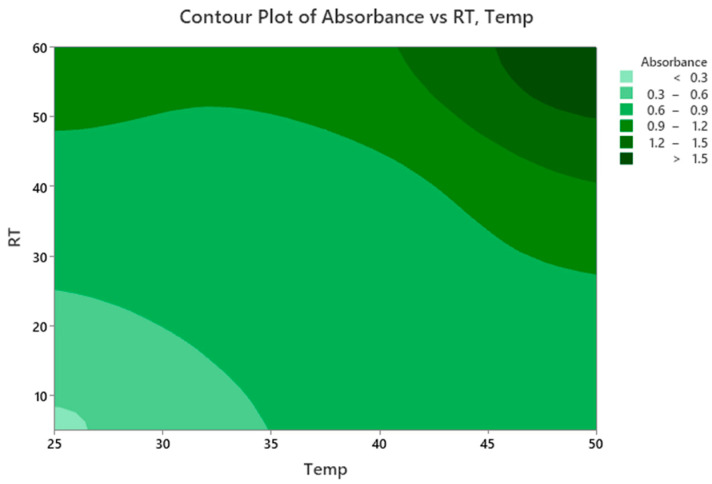
A two-dimensional contour plot for PBD of absorbance versus RT and Temp.

The contour plots, Figure 5, display the relationship between two factors and the response surface, which appears as contours. Figure 5 shows a sample contour plot for the relationship between the reaction time (RT) and the temperature (Temp), and the absorbance at 843 nm. The legend on the right-hand side shows that dark green zones are the zones in which absorbance exceeds 1.5. This absorbance value was attained using a factorial combination of RT: 53–60 min and by heating samples the at 47–50 °C.

#### 2.3.5. Optimization Phase: Individual Desirability Function

An optimization plot (desirability function plot; Figure is not shown) was utilized to find the optimum conditions that maximize the absorbance. The obtained individual desirability (*d*) was 1.0000, indicating that the optimal conditions are favorable to obtain the maximum absorbance of the CTC. The optimum conditions (which will be used for construction of calibration curve) to obtain an absorbance value of 1.000 are 25 °C, 0.50 mL of TCNQ, 60 min as RT, and ACN as DS.

### 2.4. Validation of the Proposed Method

#### 2.4.1. Linear Range and Sensitivity

The calibration curve of the CTC formation reaction shows a linear relationship between the concentration (0.5–10 µg mL^−1^) and absorbance recorded at λ_max_ = 843 nm. The linear relationship is presented by Equation (3):

Y = 0.1132x + 0.0611,  R^2^ = 0.9975 (3)

The value of R^2^ was relatively high, reflecting the linearity of obtained data. The analytical data are summarized in Table 5. The sensitivity was assessed using the limit of detection (LOD) and limit of quantification (LOQ), determined using Equations (4) and (5):

(4) $$\mathrm{LOD}=3\times \frac{SD}{a}$$

(5) $$\mathrm{LOQ}=10\times \frac{SD}{a}$$

where *SD* represents the standard deviation of the blank and *a* is the slope of the straight line of the calibration curve of TIGC. The LOD and LOQ values are listed in Table 5. The values of LOD and LOQ indicate that the proposed method is extremely sensitive, with a nanogram level detection limit.

#### 2.4.2. Accuracy and Precision

The accuracy and precision were measured at 95.0% CI for three concentrations of TIGC as bulk powder and in the pharmaceutical dosage form. The intra-day precision was evaluated by measuring each sample three times in the same day, while the inter-day precision was assessed over three different days. The results showed that the % relative standard deviation (%RSD) was ˂3%, indicating good precision. The accuracy was expressed in terms of %RE. Table 6 summarizes the obtained data and shows the accuracy and precision of the current method. The accuracy was additionally evaluated by applying the standard addition technique, as shown in Table 6. Calibration and standard addition methods were applied to the pharmaceutical formulation and the %mean recovery values for both methods were high (100.50 ± 2.91% for the calibration method and 100.80 ± 2.46% for the standard addition method), as shown in Table 6, indicating that the matrix (commonly added adjuvants and diluents) has a minor effect.

#### 2.4.3. Comparison to the Reference Method

The proposed method was compared to a reference spectrophotometric method in terms of the student *t*-test and an F-statistic, as shown in Table 7. The results show that the obtained *t*- and *F*- values were less than the tabulated values signifying that the proposed technique is comparable to the reference approach [17].

#### 2.4.4. Determination of the Reaction Stoichiometry

The molecular composition of the resultant CTC was determined by conducting Job’s method of continuous variation. A plot of the relationship between the absorbance of the resulting complex versus the mole fraction (V_r_/V_r_ + V_d_) was drawn, where V_r_ is the volume of TCNQ and V_d_ is the volume of TIGC—the Figure is not shown. The maximum amount of the complex formed was observed at a mole fraction of 0.50; therefore, the best stoichiometric ratio for the reaction of TIGC and TCNQ was 1:1.

### 2.5. Investigation of the Reaction Thermodynamics

**Figure 6:**
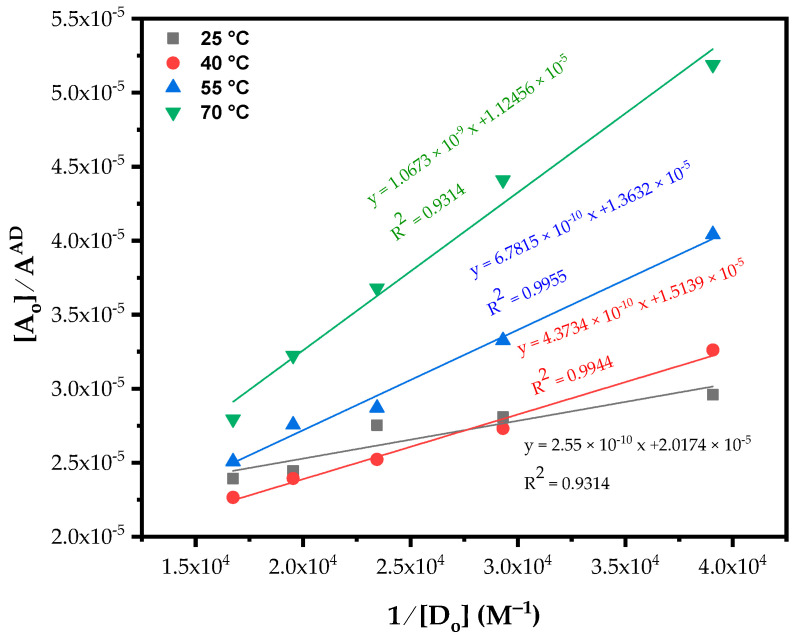
Benesi–Hildebrand plots at temperatures in the range of 25–70 °C.

The formation constant for the charge transfer reaction of TIGC and TCNQ was calculated using the Benesi–Hildebrand equation [40,41,42,43] as follows (Equation (6)):

(6) $$[A_{o}]/A^{AD}=1/\varepsilon^{AD}+1/\varepsilon^{AD}K_{c}^{AD}\times 1/\left[D_{o}\right]$$

where [*A*_o_] and [*D*_o_] denote the initial concentrations of TCNQ and TIGC, respectively, and *A^AD^, ε^AD^*, and $K_{c}^{AD}$ are the absorbance, the molar absorptivity, and the formation constant of the CTC, respectively. The Benesi–Hildebrand equation can be applied for the 1:1 CTC by keeping [*A*_o_] lower than [*D*_o_]. In other words, [*D*_o_] must be 5–10 times higher than [*A*_o_]. The thermodynamic parameters were obtained by studying the reaction at four different temperatures (25, 40, 55, and 70 °C). Plots of $[A_{o}]/\;A^{AD}$ versus $1/\left[D_{o}\right]$ were sketched, as shown in Figure 6. Straight-line equations were acquired for the CTC at different temperatures. The slope of the straight-line is 1/$\varepsilon^{AD}$ and the intercept is $1/\varepsilon^{AD}K_{c}^{AD}$. The obtained figures for $K_{c}^{AD}$ and *ε^AD^*, determined at 25, 40, 55, and 70 °C, are displayed in Table 8.

The obtained data show that $K_{c}^{AD}$ and *ε^AD^* at room temperature (298 K) have reached high values of 7.90 × 10^4^ L mol^−1^ and 4.96 × 10^4^ L mol^−1^ cm^−1^, thus corroborating the stability of the formed CTC. These high values could be ascribed to the high donating ability of TIGC, prominent electron affinity of TCNQ, and the high electric permittivity of ACN. Moreover, these high values demonstrate the dissociation of the outer sphere CTC (TIGC-TCNQ) resulting in the formation of radical ions of the two species with high electrostatic attraction, as shown in Scheme 3 [44,45,46,47].

**Figure 7:**
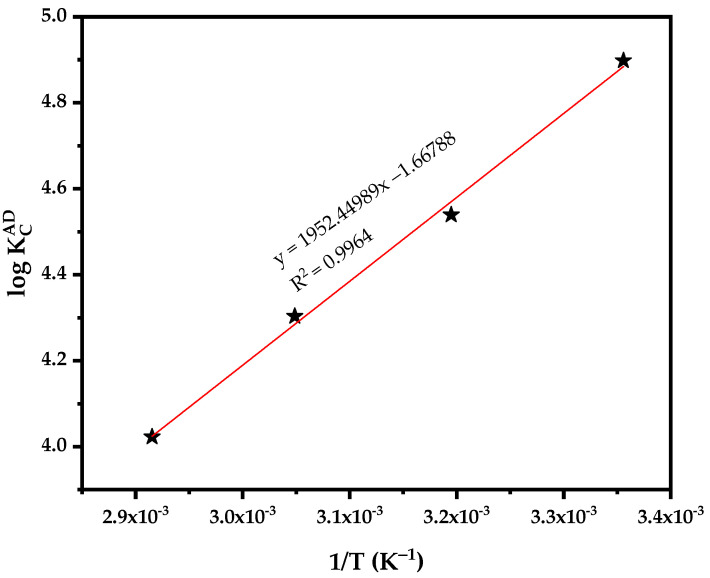
Van’t Hoff plot for the TIGC-TCNQ complex.

According to the obtained data at the different temperatures, the complex formation was thermodynamically favored as reflected by the immense negative value of the standard Gibbs free energy change (∆*G°*), ranging between −26.42 and −27.95 kJ/mol. The value of ∆*G°* decreases (less negative) as the temperature increases, implying that a complex formation might not be favored at a higher temperature. The formation constant values, $K_{c}^{AD},$ confirm these findings. The values of $K_{c}^{AD},$ calculated at 298, 313, 328, and 343 K, were employed to compute the standard enthalpy of formation (∆*H°*) employing Van’t Hoff plots as depicted in Figure 7 (summary is shown in Table 8) and utilizing Equation (7) [48,49]:

(7) $$\log\;K_{c}^{AD}=-∆H^{{}^{\circ}}/2.303\;\mathrm{RT}+\mathrm{constant}$$

In this equation, R denotes the gas constant (8.314 J/mol. K), and T stands for the temperature (Kelvin). Plotting log $K_{c}^{AD}$ against the reciprocal of the absolute temperature, as shown in Figure 7, has resulted in a linear relationship with a slope of −∆*H°*⁄(2.303 R).

The determined ∆*H°* is a high negative value, signifying that the formation of the TIGC-TCNQ CTC is exothermic in nature. The standard entropy change (∆*S°*) was obtained using Equation (8):

∆*G*° = ∆*H*° − T∆*S*° (8)

The value of ∆*S°* was low (−31.93 ± 0.37 J/K. mol), indicating the simple composition of the CTC without the interference of the solvent.

**Figure 8:**
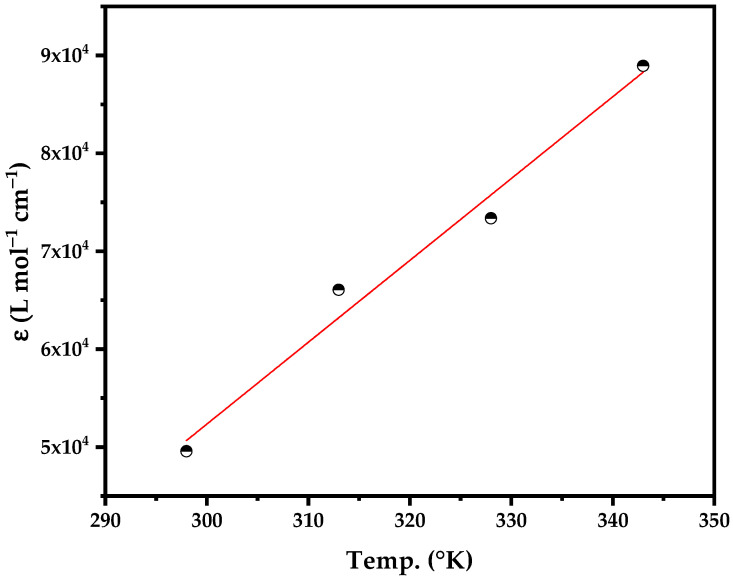
The relationship between the temperature (K) and the molecular extinction coefficient (ε).

Table 8 also shows that the value of *ε^AD^* (molecular extinction coefficient) increases as the temperature increases, as shown in Figure 8. Conversely, the value of *ε^AD^* is inversely proportional to the formation constant, $K_{c}^{AD}$.

### 2.6. Evaluation of the Reaction Kinetics

The kinetic studies were conducted by measuring the absorbance of the resultant CTC after reaction times of 5, 15, 30, 45, 60, 75, and 90 min. The obtained data show that the absorbance of the CTC increases as the reaction time increases. This behavior was utilized to assess the reaction kinetics. Employing the formerly outlined optimum settings, the initial rates were obtained from the slopes of absorbance–time graphs. The reaction order was then established corresponding to the donor reactant (TIGC) in the presence of a fixed concentration of acceptor (TCNQ). In this study, several kinetic models were used, including the initial rate, rate constant, fixed concentration, and fixed time approaches [50,51,52,53,54]. Moreover, these techniques were evaluated, and the choice of the best technique depended merely on the relevance, LOD, LOQ, and linear range of the acquired data.

#### 2.6.1. Initial Rate Approach

Following the previously outlined optimal conditions, the reaction’s initial rates were established by utilizing the slopes of the absorbance–time plots. The reaction order can be determined by plotting reaction rates versus the initial absorbance. Consequently, the rate of reaction can be represented by Equation (9):

Rate = K′[Acceptor]^m^[Donor]^n^ (9)

In this equation, K′ symbolizes the rate constant, and m + n signifies the overall reaction order with respect to TCNQ (m) and TIGC (n). The initial rate of the CTC formation reaction follows a pseudo-first-order reaction rate and can be expressed as follows, in Equation (10):

Log (rate) = log K = logΔA/Δt = log k′ + n log [C](10)

In this equation, A represents the absorbance and *t* is the reaction time (s). A regression of the log (rate) versus log [TIGC] is provided Equation (11):

Log (rate) = log K′ = log ΔA/Δt = 1.1742 + 0.9977 log C, R^2^ = 0.9526 (11)

Therefore, K′ = 14.93 s^−1^ and the slope = n = 0.9977 ≃ 1, substantiating that the CTC formation follows the pseudo-first-order. Based on these data, the reaction rate was [TIGC]-dependent and would be best portrayed using Equation (12):

Rate = K′[TIGC]^n^ (12)

In Equation (12), K′ represents the pseudo-first-order rate constant, and n denotes the reaction order related to [TIGC].

#### 2.6.2. Fixed-Time Approach

In this approach, calibration curves were drawn in the range of 1–8 µg mL^−1^ of TIGC at reaction times of 5, 15, 30, 45, 60, 75, and 90 min. The regression equations for each reaction time were calculated and the results are shown in Table 9. From the results, the best linearity was obtained at 60 min, confirming the findings of the optimization phase of the PBD.

#### 2.6.3. Rate Constant Approach

Log A against time (s) plots were obtained for concentrations of 1–8 µg mL^−1^ (1.71 × 10^−6^ −1.36 × 10^−5^ M) of TIGC. A plot of K′ versus concentration (M) was then plotted, and Equation (13) was obtained:

K′ = 33.986x − 0.0008, R^2^ = 0.8492 (13)

#### 2.6.4. Fixed Concentration Approach

Graphs of absorbance versus time (min) were plotted and a straight line was depicted to intersect with the largest possible number of curves. Another graph of 1/t (s) versus concentration (M) was thenceforth obtained, Equation (14):

1/t = 0.0002 C − 0.0007, R^2^ = 0.9798 (14)

#### 2.6.5. Assessment of the Kinetics Procedures

A comparison between the four procedures proposed for the determination of the reaction kinetics is shown in Table 10. It is evident that the initial rate and fixed time methods showed the widest linear range with the lowest LOD and LOQ. Yet, the rate constant method shows less linearity compared to the ones shown for the fixed-time and fixed-concentration methods.

## 3. Materials and Methods

### 3.1. Instrumentation and Software

The software employed to constitute the PBD matrix was Minitab^®^19 (Minitab^®^ Inc., State College, PA, USA). Deionized water utilized in the current investigation was obtained from a Millipore-Q water system (Burlington, MA, USA). Absorbance was determined by means of a UV-Vis spectrophotometer (Agilent diode-array, Agilent, Santa Clara, CA, USA) equipped with 10 mm quartz cuvettes (matched). Samples were heated to the required temperatures when needed using a thermostatically controlled water bath.

### 3.2. Reagents and Standards

Stock solutions of TIGC (0.05%) and TCNQ (0.1%) were freshly prepared daily by weighing specific amounts of TIGC and TCNQ powders and diluting them with acetonitrile to the mark in 100 mL volumetric flasks. Working solutions were obtained via serial dilutions of the stock solutions using the same solvent.

### 3.3. Materials

Tigecycline (TIGC, Batch# AT108181901) was the product of Biosynth^®^ Carbosynth Ltd. (Compton, Berkshire, UK). The electron acceptor 7,7,8,8-tetracyanoquinodimethane (TCNQ, purity 98%) was purchased from Sigma-Aldrich (St. Louis, MO, USA). Acetonitrile procured from BDH Chemicals (BDH Laboratory Supplies, Poole, UK) and methanol from Merck KGaA (Darmstadt, Germany) were used as received with no further purification. Tygacil^®^ vials were purchased from local pharmacy stores in Cairo, Egypt.

### 3.4. General Procedures

#### 3.4.1. Authentic Samples: Design of Experiments (DoE)

The studied variables and their proposed levels are listed in Table 1. Twenty-four experimental runs took place (with 4 center points). Aliquots of 0.05% stock solution (500 µg mL^−1^ of TIGC) were used in all experimental runs. A volume of 0.1% TCNQ, as shown in the scenario revealed in Table 2, was added to TIGC solution and volume was completed to the mark using the suitable diluting solvent. Type of diluting solvent, temperature at which reaction took place, and the reagent volume are all shown for each run in the design matrix table, as shown in Table 2. Reagent blanks were prepared and measured similarly. The absorbance of the resulting green colored solutions was measured at λ_max_ = 843 nm. To create the calibration curve, different concentrations of the stock solution were prepared from the TIGC stock solutions by serial dilution to obtain a final concentration in the range of 0.5–10 µg mL^−1^, and the calibration curve was constructed using the same procedure and implementing the optimal conditions.

#### 3.4.2. Procedure for the Formulation

Tygacil^®^ vials (product of Patheon Italia S.P.A., Italy), labelled to contain 50 mg TIGC per vial, were the formulation of choice. The lyophilized content of the vial was further crushed and an amount of 30.8 mg of the powdered material (equivalent to 10.0 mg TIGC) was accurately weighed, dissolved in ACN, filtered, and transferred into a 50 mL volumetric flask. The volume was completed using ACN and formulation stock solution of 200 µg mL^−1^ TIGC was then ready for further analysis.

#### 3.4.3. Standard Addition Method

Ten samples were made by inserting a fixed amount of 30 µL (200 µg mL^−1^) of the formulated drug solution to ten volumetric flasks (labelled as S0–S10). Different volumes (10–180 µL) of TIGC (0.05%) were then added followed by applying the optimum conditions and measuring the absorbance at λ_max_ = 843 nm.

#### 3.4.4. Procedure for Job’s Method

Job’s method [27] was applied to ascertain the molar ratio for the interaction of TIGC with TCNQ. Equimolar solutions (0.854 mM) of TIGC and TCNQ were prepared. A total of ten samples (10 mL) were prepared where the total volume was kept at 2.0 mL of both drug and reagent for each solution, with the aid of acetonitrile as a solvent. Absorbance of the prepared set was recorded at λ_max_ = 843 nm contrasted to a reagent blank.

#### 3.4.5. Evaluation of the Thermodynamic Parameters

Samples were prepared by keeping [TIGC] at least five times that of >[TCNQ], [D] >> [A], where D is the electron donor and A is the electron acceptor. Four sets with five samples each were prepared in volumetric flasks (10 mL). Volumes (1–3 mL) of 0.85 mM TIGC were inserted. Volume of 1.0 mL of 0.17 mM of TCNQ was then added. Solutions were then inserted in water bath for 60 min at 25, 40, 55, and 70 °C, followed by a measurement of the absorbance at 843 nm.

#### 3.4.6. Investigation of the Reaction Kinetics

Seven sets were prepared with a [TIGC] of 1–8 µg mL^−1^. Absorbance of every group was recorded at 5, 15, 30, 45, 60, 75, and 90 min as described under the general procedure.

## 4. Conclusions

A simple, sensitive, and selective methodology was developed to generate a CTC involving tigecycline as the electron donor and TCNQ as a π- acceptor. The Plackett–Burman Design (PBD) was implemented to attain the optimum process variables and the maximum response—absorbance of the CTC in this case. A Pareto chart and ANOVA testing were used to determine the statistical significance of the studied variables. The results showed that the RV was not statistically significant. The desirability function plot showed that the optimum conditions that could be used to maximize the response were Temp: 25 °C, RV: 0.50 mL, RT: 60 min, and ACN as DS. Job’s method of continuous variation showed that the complex has a molecular composition of 1:1. ICH recommendations were used to assess the developed technique. The obtained data show excellent accuracy and precision with no significant differences compared to the reference method. Interferences from the common excipients and additives were not observed. Therefore, the developed approach could be utilized for the routine analysis of tigecycline in its pure form and in formulations. The calibration curve obtained under the optimum conditions was rectilinear in the range 0.5–10 µg mL^−1^. The stability of the CTC complex was determined using thermodynamic studies; thus, the Benesi–Hildebrand equation and Van’t Hoff plots showed the formation of a stable complex with a formation constant of 1.05 × 10^4^ − 7.90 × 10^4^ L mol^−1^ and a molar absorptivity of 4.96 × 10^4^ − 8.89 × 10^4^ L mol^−1^ cm^−1^. The Reaction kinetics were studied and revealed that the interaction between TIGC and TCNQ follows a pseudo-first-order reaction, and the best linearity was obtained at RT: 60 min.

## Figures and Tables

**Table 1:** Variables affecting CTC formation reaction. Measured response (Y_ctc_) is absorbance at 843 nm.

Variables	Code	Minimum (−)	Central (0)	Maximum (+)
**Independent—Numerical**				
Temperature (Temp, °C)	A	25	37.5	50
Reagent volume (RV, mL)	B	0.5	1.0	1.5
Reaction time (RT, min)	C	5	32.5	60
**Independent—Non-numerical**				
Diluting solvent (DS)	D	Methanol	Acetonitrile	

**Table 2:** Design matrix: the observed and predicted responses.

		Independent Variables			Dependent Variables			
Run Order	Pattern of Investigated Variables	A ^a^ (°C)	B ^b^ (mL)	C ^c^ (min)	D ^d^	Y_ctc_ Obs *	Y_ctc_ Pred *	RE **
1	+ + + −	50	1.5	60	Methanol	1.633	1.565	0.043
2	− + + +	25	1.5	60	Acetonitrile	1.122	1.099	0.02
3	+ − + +	50	0.5	60	Acetonitrile	1.787	1.736	0.029
4	− − − −	25	0.5	5	Methanol	0.262	0.203	0.29
5	− − + +	25	0.5	60	Acetonitrile	1.073	1.004	0.069
6	− + − +	25	1.5	5	Acetonitrile	0.311	0.372	0.164
7	− − + −	25	0.5	60	Methanol	0.765	0.79	0.032
8	− + + −	25	1.5	60	Methanol	0.764	0.875	0.129
9	+ + − −	50	1.5	5	Methanol	0.665	0.661	0.006
10	+ + + +	50	1.5	60	Acetonitrile	1.659	1.862	0.109
11	+ − − +	50	0.5	5	Acetonitrile	0.733	0.773	0.052
12	0 0 0 +	37.5	1	32.5	Acetonitrile	0.822	0.845	0.027
13	− − − +	25	0.5	5	Acetonitrile	0.311	0.318	0.022
14	+ + − +	50	1.5	5	Acetonitrile	0.947	0.858	0.104
15	0 0 0 −	37.5	1	32.5	Methanol	0.795	0.65	0.223
16	− − − −	25	0.5	5	Methanol	0.169	0.203	0.167
17	+ + − −	50	1.5	5	Methanol	0.721	0.661	0.091
18	− + + −	25	1.5	60	Methanol	0.972	0.875	0.111
19	0 0 0 −	37.5	1	32.5	Methanol	0.468	0.649	0.279
20	+ − − −	50	0.5	5	Methanol	0.523	0.587	0.109
21	0 0 0 +	37.5	1	32.5	Acetonitrile	0.937	0.845	0.109
22	− + − +	25	1.5	5	Acetonitrile	0.392	0.372	0.054
23	+ − + −	50	0.5	60	Methanol	1.488	1.451	0.025
24	+ − + +	50	0.5	60	Acetonitrile	1.723	1.736	0.007

^a,b,c,d^ Coded variables are as outlined in Table 1, * Y_CTC_(Obs): Observed absorbance values of the CTC at λ_max_ = 843 nm, ** Y_CTC (Pred)_: Predicted absorbance values following response transformation, ** RE: Relative error calculated as RE = | (Experimental Value—Predicted Value)/Predicted Value |.

**Table 3:** Analysis of variance (ANOVA) test.

Source	DF *	Adj SS *	Adj MS *	F-Value *	*p*-Value *
Model	5	1.55413	0.310826	103.81	0.000
Linear	4	1.54743	0.386857	129.20	0.000
Temp	1	0.49894	0.498938	166.63	0.000
RV	1	0.01090	0.010896	3.64	0.073
RT	1	0.96063	0.960633	320.82	0.000
DS	1	0.07696	0.076961	25.70	0.000
Curvature	1	0.00670	0.006704	2.24	0.152
Error	18	0.05390	0.002994		
Lack-of-Fit	11	0.01598	0.001453	0.27	0.974
Pure Error	7	0.03792	0.005417		
Total	23	1.60803			

* DF is degrees of freedom; SS is sum of squares, and MS is mean of squares. Adj SS is the adjusted sum of squares, and Adj MS is the adjusted mean of squares.

**Table 4:** Descriptive statistic for the 2-sample *t*-test. The table footnote shows the used hypotheses and the associated statistical parameters.

Solvent Used	N	Mean	SD	SE Mean	*p*-Value
Methanol	12	0.769	0.436	0.130	0.285
Acetonitrile	12	0.985	0.524	0.150	

Null hypothesis: H_0_: μ_1_ − µ_2_ = 0, Alternative hypothesis: H_1_: μ_1_ − µ_2_ ≠ 0, T-Value = −1.10, DF = 21.

**Table 5:** Analytical parameters for the determination of TIGC-TCNQ CTC at λ_max_ = 843 nm.

Parameter	Value
Linear range (μg mL^−1^)	0.5–10
Molar absorptivity (L mol^−1^ cm^−1^)	6.63 × 10^4^
Regression equation (y = *a*x + *b*)	
Slope (a)	0.1132
Intercept (b)	0.0611
Coefficient of determination (R^2^)	0.9975
LOD (ng mL^−1^)	166
LOQ (ng mL^−1^)	504

**Table 6:** Determination of TIGC as per se and in Tygacil^®^ vials operating the optimum conditions. Inter- and intra-day precision assays are shown.

**Determination of TIGC in Pure form**		
**Taken (µg mL^−1^)**	**Found (µg mL^−1^)**	% Recovery *
0.50	0.49	97.67
1.00	0.96	96.36
2.00	2.07	103.7
4.00	3.88	97.02
6.00	6.14	102.3
8.00	8.02	100.3
10.0	9.94	99.41
Mean * ± SD	99.53 ± 2.74	
RSD	2.75	
**Determination of TIGC in Tygacil^®^ Vials: Direct Calibration Method**		
**Taken (µg mL^−1^)**	**Found** **(µg mL^−1^)**	% Recovery *
0.50	0.51	102.72
1.00	1.00	100.08
2.00	2.08	103.99
4.00	3.79	94.86
6.00	6.09	101.57
8.00	8.03	100.43
10.0	9.98	99.81
Mean * ± SD	100.50 ± 2.91	
RSD	2.89	
**Determination of TIGC in Tygacil^®^ Vials: Standard Addition Method**		
**Taken (µg mL^−1^)**	**Added** **(µg mL^−1^)**	% Recovery *
1.00	0.00	97.53
1.00	0.50	103.19
1.00	1.00	103.52
1.00	1.50	98.39
1.00	2.00	103.63
1.00	2.50	97.75
1.00	3.00	102.09
1.00	3.50	100.56
1.00	4.00	100.92
Mean * ± SD	100.80 ± 2.46	
RSD	2.44	
**Precision Assays (Inter- and Intra-Day) of TIGC in Pure Form**		
**Concentration (µg mL^−1^)**	Mean % Recovery *±SD	**Error (%)**
(a) Inter-day		
1.00	98.37 ± 1.58	1.63
4.00	99.75 ± 0.66	0.26
10.0	99.83 ± 1.04	0.17
(b) Intra-day		
1.00	99.33 ± 3.21	0.67
4.00	99.50 ± 0.50	0.50
10.0	99.67 ± 1.15	0.33
**Precision Assays (Inter- and Intra-Day) of TIGC in Tygacil^®^ Vials**		
**Concentration (µg mL^−1^)**	Mean % Recovery * ± SD	**Error (%)**
(a) Inter-day		
2.00	99.00 ± 1.52	1.00
6.00	100.17 ± 1.32	0.17
10.0	100.33 ± 0.76	0.67
(b) Intra-day		
2.00	101.17 ± 1.51	1.17
6.00	100.22 ± 1.26	0.22
10.0	101.10 ± 0.85	1.10

* Mean ± SD of three assays.

**Table 7:** A comparison between the proposed and the reported [17] methods for the determination of TIGC using TCNQ in the pure form and in the vials.

Parameter	Proposed Method	Reported Method	Tygacil^®^ Vials
Mean %Recovery *	99.53	99.10	100.5
±SD	2.74	1.33	2.91
RSD	2.75	1.34	2.89
V	7.51	1.77	8.45
n	7	3	7
±SE	1.04	0.77	1.19
*t*	0.253 (2.306) ^a^		
F	4.24 (19.3) ^b^		

* Average of three assays, ^a,b^ the tabulated t-values and F-ratios at *p* = 0.05 are shown between parentheses.

**Table 8:** Thermodynamic parameters for the determination of TIGC using CTC formation at various temperatures, λ_CTC_ is 843 nm.

Temp (K)	$K_{c}^{AD}\;(L\;{\mathrm{mol}}^{-1})$	*ε* (L mol^−1^ cm^−1^)	∆*G°* (kJ/mol)	∆*H*° (kJ/mol)	∆*S°* (J/K mol)
298	7.90 × 10^4^	4.96 × 10^4^	−27.95	−37.38	−31.93 ± 0.37
313	3.46 × 10^4^	6.61 × 10^4^	−27.20		
328	2.01 × 10^4^	7.34 × 10^4^	−27.03		
343	1.05 × 10^4^	8.89 × 10^4^	−26.42		

**Table 9:** Regression equations obtained at fixed times of 5, 15, 30, 45, 60, 75, and 90 min for the determination of TIGC employing CTC formation with TCNQ.

Time (min)	Regression Equation	R^2^ Value
5	A = 0.0398C + 0.0032	0.8850
15	A = 0.0607C + 0.0318	0.9921
30	A= 0.0725C + 0.0060	0.9915
45	A = 0.0940C + 0.0403	0.9933
60	A = 0.1131C + 0.0677	0.9989
75	A = 0.0933C + 0.0976	0.9921
90	A = 0.1059C + 0.0671	0.9955

**Table 10:** Regression parameters for the approached kinetics methods.

Parameter	Initial Rate Approach	Fixed-Time Approach	Rate Constant Approach	Fixed Concentration Approach
Linear range (µg mL^−1^)	1–8	1–8	4–8	4–8
S_b_	0.1112	0.0017	1.7288 × 10^−5^	2.2900 × 10^−5^
±tS_b_	0.0770	0.0012	1.3833 × 10^−5^	8.9147 × 10^−6^
S_a_	0.5816	0.0083	1.0264 × 10^−4^	1.3592 × 10^−4^
±tS_a_	0.4030	0.0057	8.2129 × 10^−5^	5.2928 × 10^−5^
S_y/x_	0.5634	0.4192	2.9465 × 10^−8^	4.4453 × 10^−7^
LOD (µg mL^−1^)	0.2768	0.2846	2.9081	0.9914
LOQ (µg mL^−1^)	0.8387	0.8625	8.8125	3.0040

S_b_ = SD of slope, ±tS_b_ = confidence limit for slope, S_a_ = SD of intercept, ±tS_a_ = confidence limit for intercept, S_y/x_ = SD of the regression.

## Data Availability

Data is contained within the article.
